# Deadly diving? Physiological and behavioural management of decompression stress in diving mammals

**DOI:** 10.1098/rspb.2011.2088

**Published:** 2011-12-21

**Authors:** S. K. Hooker, A. Fahlman, M. J. Moore, N. Aguilar de Soto, Y. Bernaldo de Quirós, A. O. Brubakk, D. P. Costa, A. M. Costidis, S. Dennison, K. J. Falke, A. Fernandez, M. Ferrigno, J. R. Fitz-Clarke, M. M. Garner, D. S. Houser, P. D. Jepson, D. R. Ketten, P. H. Kvadsheim, P. T. Madsen, N. W. Pollock, D. S. Rotstein, T. K. Rowles, S. E. Simmons, W. Van Bonn, P. K. Weathersby, M. J. Weise, T. M. Williams, P. L. Tyack

**Affiliations:** 1 Sea Mammal Research Unit, Scottish Oceans Institute, University of St Andrews, Fife KY16 8LB, UK; 2 Department of Life Sciences, Texas A&M University-Corpus Christi, Corpus Christi, TX 78412, USA; 3 Department of Biology, Woods Hole Oceanographic Institution, Woods Hole, MA 02543, USA; 4 Department of Animal Biology, La Laguna University, 38256 Tenerife, Canary Islands, Spain; 5 Institute of Animal Health, University of Las Palmas de Gran Canaria, 35413 Arucas, Las Palmas, Spain; 6 Department of Circulation and Medical Imaging, Norwegian University of Science and Technology, Trondheim, NO-7491, Norway; 7 Department of Ecology and Evolutionary Biology, University of California, Santa Cruz, CA 95064, USA; 8 Aquatic Animal Health Program & Department of Physiological Sciences, College of Veterinary Medicine, University of Florida, Gainesville, FL 32610, USA; 9 The Marine Mammal Center, Sausalito, CA 94965, USA; 10 Charité, Universitätsmedizin Berlin, D-13353 Berlin, Germany; 11 Brigham and Women's Hospital and Harvard Medical School, Boston, MA 02115, USA; 12 Department of Physiology and Biophysics, Dalhousie University, Halifax, Canada B3H 4H7; 13 Northwest ZooPath, Monroe, WA 98272, USA; 14 Biomimetica, Santee, CA 92071, USA; 15 Institute of Zoology, Regents Park, London, NW1 4RY, UK; 16 Harvard Medical School, Boston, MA 02115, USA; 17 Norwegian Defense Research Establishment (FFI), Horten, NO-3191, Norway; 18 Zoophysiology, Department of Bioscience, Aarhus University, 8000 Aarhus C., Denmark; 19 Divers Alert Network & Duke University Medical Center, Durham, NC 27710, USA; 20 National Oceanic and Atmospheric Administration, Silver Spring, MD 20910, USA; 21 Marine Mammal Commission, Bethesda, MD 20814, USA; 22 Gales Ferry, CT 06335, USA; 23 Office of Naval Research, Marine Mammals & Biological Oceanography Program, Arlington, VA 22203, USA

**Keywords:** diving physiology, marine mammals, gas bubbles, embolism, decompression sickness

## Abstract

Decompression sickness (DCS; ‘the bends’) is a disease associated with gas uptake at pressure. The basic pathology and cause are relatively well known to human divers. Breath-hold diving marine mammals were thought to be relatively immune to DCS owing to multiple anatomical, physiological and behavioural adaptations that reduce nitrogen gas (N_2_) loading during dives. However, recent observations have shown that gas bubbles may form and tissue injury may occur in marine mammals under certain circumstances. Gas kinetic models based on measured time-depth profiles further suggest the potential occurrence of high blood and tissue N_2_ tensions. We review evidence for gas-bubble incidence in marine mammal tissues and discuss the theory behind gas loading and bubble formation. We suggest that diving mammals vary their physiological responses according to multiple stressors, and that the perspective on marine mammal diving physiology should change from simply *minimizing N*_2_ *loading* to *management of the N*_2_ *load*. This suggests several avenues for further study, ranging from the effects of gas bubbles at molecular, cellular and organ function levels, to comparative studies relating the presence/absence of gas bubbles to diving behaviour. Technological advances in imaging and remote instrumentation are likely to advance this field in coming years.

‘The question of bends in diving mammals keeps rising from corpses of the deep, and will continue to do so because it is such an intractable experimental problem.’ [1, p. 516]

## 1. Introduction

The effects of hydrostatic pressure can cause a plethora of challenges related to the management of nitrogen gas (N_2_) for divers. Under pressure, lung gases in diving vertebrates move to the blood and other tissues of the body according to gas tension gradients and perfusion levels [2,3]. As hydrostatic pressure increases with depth, the amount of N_2_ that is absorbed by the blood and tissues increases, resulting in higher dissolved gas tensions that would maximally reach equilibrium with the partial pressure of N_2_ in the lungs. This is a long-known problem for human divers breathing pressurized air, but has often been discounted as a problem for breath-hold divers since they dive on only a single inhalation. However, for free-diving animals, tissues can become highly saturated under certain circumstances depending on the iterative process of loading during diving and washout at the surface [4]. During decompression, if the dissolved gas tension in the tissues cannot equilibrate fast enough with the reducing partial pressure of N_2_ in the lungs, tissues will become supersaturated, resulting in the potential for gas-bubble formation. Although bubbles can form without negatively impacting a diving animal (i.e. ‘silent bubbles’), N_2_ gas emboli formation is generally held to be a pivotal event in the occurrence of decompression sickness (DCS) [5].

**Figure 1.**
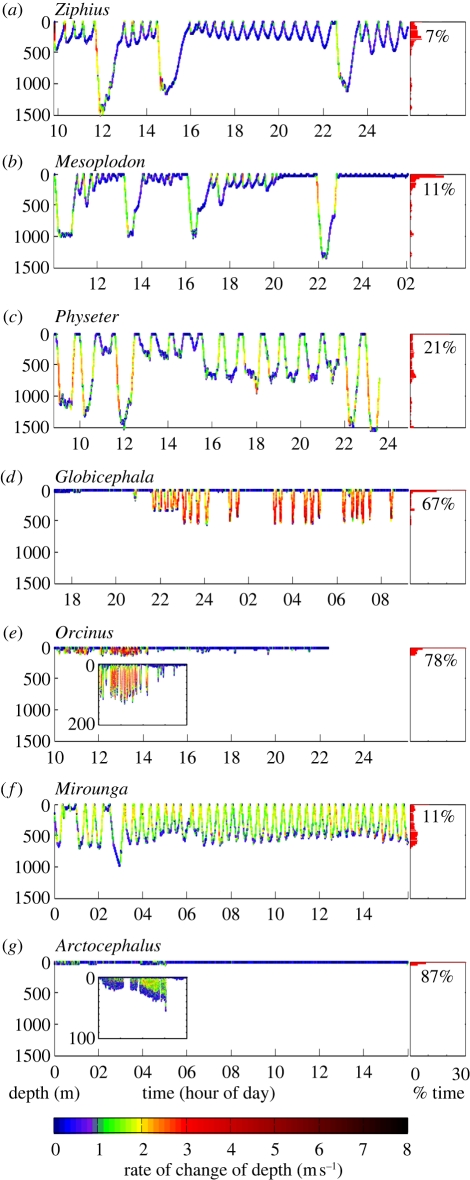
Variability in diving behaviour of a range of marine mammal species. Dive traces are plotted to identical scales: 1500 m depth over a 16 h time period for each of: (*a*) Cuvier's beaked whale (*Ziphius*), (*b*) Blainville's beaked whale (*Mesoplodon*), (*c*) sperm whale (*Physeter*), (*d*) pilot whale (*Globicephala*), (*e*) killer whale (*Orcinus*), (*f*) northern elephant seal (*Mirounga*) and (*g*) Antarctic fur seal (*Arctocephalus*). Traces are coloured according to vertical speed (rate of change in depth). Insets to only 200 and 100 m depth are shown for 4 h portions of *Orcinus* and *Arctocephalus* plots, respectively. Histograms show percentage of time spent at depth (10 m bins, from 0 to 1500 m), with numerical display of percentage time at 0–10 m. Data sources: WHOI Dtag group (*a*–*e*), D. Costa (*f*), S. Hooker (*g*).

Nevertheless, marine mammals dive routinely and repeatedly to substantial depths without apparent injury (figure 1). Studies over the past half-century have suggested that this is due to anatomical, physiological and behavioural adaptations to prevent the formation of gas emboli in blood and other tissues [6–8] (see also electronic supplementary material, table S1). Fundamental to blood N_2_ kinetics is its uptake at the blood–lung interface. Scholander [6] first proposed that the stiffened upper airways of marine mammals (with lungs lacking smaller branching respiratory bronchi) would receive air from more compressible airways during descent. A progressive collapse of alveoli was thought to prevent gas uptake by the blood beyond some critical depth of lung collapse, thus limiting the amount of N_2_ that was absorbed on a dive. Further, all vertebrates possess to varying degrees an autonomic reflex known as the ‘diving response’, which functions to conserve oxygen stores and hence prolong maximum dive times [6], but will also limit N_2_ uptake. This reflex is well developed in diving marine mammals, birds and reptiles and is manifested as peripheral vasoconstriction (reduced blood flow to the muscles) and associated bradycardia (reduced heart-rate). Early work with captive animals in forced dives showed a profound response (heart rate declining from 150 beats per minute to 10 beats per minute [6]). This was confirmed in several field studies, though the effect was considerably more variable with unrestrained animals [9–13]. Several anatomical and physiological traits (such as increased body mass, decreased relative lung size, increased blood volume and increased myoglobin concentration) are also found for deeper- and longer-diving species, suggesting they are dive-related adaptations (electronic supplementary material, table S1).

These observations, together with the fact that marine mammals, birds and reptiles dive on breath-hold rather than breathing compressed air at depth, led to the assumption that the likelihood of decompression-related injury was much reduced. We review several sources of evidence suggesting emboli formation in marine mammals that challenge this view. Fatalities associated with emboli appear rare, recorded primarily for beaked whales in association with anthropogenic activities such as military sonar or seismic surveys [14]. Curtailing such anthropogenic activities would be one strategy to avoid potential injury or deaths associated with pathological bubble formation in marine mammals. However, a more comprehensive understanding of the behavioural and physiological mechanisms underlying the formation and detrimental effects of gas bubbles in marine mammals would enable a more informed approach to mitigation of anthropogenically-induced diving injury [15]. This review outlines current understanding of the mechanisms thought to lead to DCS, the evidence for bubble-related injuries in marine mammals and current knowledge of physiological adaptations to diving in these species. We conclude that marine mammals may deal with bubbles on a more regular basis than previously thought and present suggestions for further work on this topic.

## 2. The effects of pressure

### (a) Human diving medicine: decompression sickness, bubble emboli and supersaturation

DCS in humans can occur when the body is subjected to sudden or rapid pressure reduction and most commonly is seen in divers, workers in compressed air chambers and aviators. Diagnosis in human divers is based on ‘*symptoms arising shortly after decompression*’ [16], including signs such as skin blotching and symptoms such as joint pain, paraesthesia, malaise, weakness and disorientation that in turn can be alleviated by immediate recompression. However, the processes leading to DCS are not well understood, owing to difficulties studying a rarely occurring illness when objective information is typically limited to ultrasonic monitoring of intravascular bubbles, with little known about events in extravascular tissues. Decompression of supersaturated tissues can result in bubble formation, which may lead to DCS either directly by ischaemia (restriction of blood supply) or indirectly by triggering biochemical cascades possibly initiating an immune response [5]. However, the thresholds and linkages between these events are not clear. Inert gas uptake and supersaturation will, at a poorly defined and variable threshold, lead to bubble formation. Gas bubbles are thought to be an important agent of DCS but other factors such as endothelial stress or insult via sensitivity to oxidative stress may also play a role [17]. Consequently, there is no clearly defined threshold for bubble quantity or size that can be related to DCS.

While the probability of DCS increases with increasing exposure pressure, exposure time and decompression rate, there can be substantial variability in its occurrence and/or symptoms. Some variability can be attributed to the dive pattern; other variations are idiosyncratic. An example of the former is that long, shallow dives tend to cause DCS-related injuries in muscles and tendons, while short, deep dives tend to involve the central nervous system [18]. In terms of individual variability, a given dive profile can generate symptoms ranging from mild to profound, and with no evident predictive factors among individuals or dive profiles.

Formation of bubbles de novo (i.e. spontaneously) requires extremely high levels of supersaturation. The presence of gas nucleation sites (e.g. cavities or lipid surfaces) reduces the pressure difference required for bubbles to form [19]. It is, therefore, commonly assumed that there are either persistent, pre-existing gas micronuclei (microscopic bubble precursors) or that nucleation of bubbles occurs at tissue interfaces *in vivo* [19].

Several risk factors, such as increasing body fat and increasing age, have been proposed to increase the likelihood of DCS, but the data are inconsistent [5]. Effects of thermal stress and exercise appear to depend on the phase of the dive in which they occur, which are probably related to their effect on perfusion. Exercise and/or tissue warming increase perfusion, increasing the absorption of inert gas as pressure increases during a dive, whereas during surfacing (decompression) increased perfusion enhances inert gas elimination and has a beneficial effect. However, the forces associated with physical exercise can also promote bubble formation [20], perhaps explaining why post-dive exercise appears to increase the risk of DCS [5]. Other factors may also play a part: release of nitric oxide (NO) or exercise at a specific time-period prior to diving appears to reduce the incidence of bubbles [21]. The working hypothesis for this effect is that bubble nuclei adhering to endothelia facilitate bubble formation. Exercise may induce protection via NO production (after a specific time-lag), which changes the properties of the vascular endothelium and reduces the possibility of bubble precursors becoming attached to the vessel walls [21].

Ultrasonic techniques, particularly audible Doppler and visual transthoracic echo imaging, have been used to study intravascular bubbles as an indicator of decompression stress in humans. These studies show there can be a substantial inter- and intra-subject variability in venous bubble formation, even after identical depth/time exposures [5]. The relationship between bubbles and DCS is also largely probabilistic, with no absolute threshold for the number or size of bubbles below which there is no risk [22]. The limitations of current technology, which drive the focus on intravascular bubbles, may confound our understanding of their significance as an agent of DCS. Emerging technologies, such as dual frequency ultrasound, should enable the study of extravascular bubbles, and may improve our understanding of the contribution of bubbles to DCS development.

While DCS for compressed gas diving has been well studied in humans, less work on DCS has involved human breath-hold divers. However, rapid, repetitive breath-hold diving in humans may also result in DCS [23]. In fact, although symptoms consistent with DCS have been more commonly reported after multiple breath-hold dives when surface intervals are short, modelling efforts have suggested that it may be possible to develop neurological DCS from a single deep breath-hold dive [24]. Among human free-divers competing to extreme depths, it is increasingly common to incorporate practices aimed to decrease the risk of DCS, including extending the surface interval between free dives to increase the opportunity for off-gassing, decompression stops during ascent and prophylactic oxygen administration post-dive.

### (b) Observation of marine mammal bubble-related diving injury

DCS was suggested following observations of lesions (i.e. abnormal tissue) coincident with intravascular and major organ gas emboli found in beaked whales mass-stranded in spatial and temporal association with military exercises deploying sonar [25,26]. The beaked whale strandings included reports of some animals behaving oddly prior to beaching. Necropsies of these cases showed several morphopathological findings, including gas bubble-associated lesions and also fat emboli in the vessels and parenchyma of vital organs [26]. These observations sparked controversy about the cause of gas emboli in such beaked whale strandings [14]. Potential mechanisms for *in vivo* bubble formation that have been considered include a direct physical effect of intense sound (such as rectified diffusion) that might destabilize gas nuclei and lead to bubble growth in N_2_ supersaturated tissues [27,28] or a behavioural change to dive profiles leading to greater-than-normal tissue supersaturation and subsequent severe gas-bubble formation [25,26,29]. Whether these observations indeed represent DCS [30] and to what degree gas bubbles may have formed after stranding [31] have been contested. However, this syndrome of massive acute gas and fat embolism has been found in animals floating offshore prior to stranding, and has not been found in beaked whale strandings from other causes of death, leading to the contention that gas and fat emboli were not caused by the stranding event itself [26].

Acute and chronic gas embolic lesions have also been reported in single-stranded cetaceans in the UK [25,29]. These severe, extensive and potentially fatal lesions reported in 10 UK-stranded cetacean species (with higher prevalence in deep-divers) had marked fibrous tissue encapsulations associated with extensive (often intravascular) gas bubbles, and so were concluded to have resulted from *in vivo* gas-bubble formation that occurred sometime prior to stranding [29].

Osteonecrosis-type surface lesions that may be interpreted as the result of chronic diving injury have been observed in sperm whale skeletal materials. These were hypothesized to have been formed by the repetitive formation of N_2_ emboli over time [32]. The diagnosis of diving-induced osteonecrosis was challenged [33] and, because of the lack of radiological or histological data, could not be definitively confirmed, although the frequency of occurrence with no apparent selective benefit is difficult to rationalize with the alternative suggestion of spondyloarthropathy. These findings suggest that sperm whales may be neither anatomically nor physiologically immune to the effects of deep diving, and that perhaps there are constraints to behaviour imposed by decompression issues. Such injury, if caused by diving, would suggest that sperm whales live with sub-lethal bubble formation on a regular basis, but with possible long-term impacts on bone health.

Bubbles have been observed from marine mammals trapped in fishing nets (by-caught), which died at depths of approximately 70–100 m [34]. The good condition of these carcasses, together with the absence of bacteria or putrefactive changes, suggests tissue gas supersaturation sufficient to cause bubbles when the animals were depressurized. Whether tissue and blood N_2_ levels represented the routine load at the time of entrapment, or whether these levels might have become elevated if the animals struggled while trapped in the net, is not clear. Recent work using B-mode ultrasound to examine the kidneys and the liver has also documented bubbles in live-stranded (common and white-sided) dolphins within minutes to hours of the stranding event [35]. Bubble presence was noted within the hepatic portal vasculature of a few animals and among the kidney renules in either the subcapsular space or vasculature of all animals, and was confirmed using CT-scanning or necropsy for animals that later died. Although bubbles were observed, animals apparently recovered or tolerated these (as confirmed by the normal behaviour recorded by satellite tags attached to released animals after the strandings).

The occurrence of DCS was suggested as the cause of death for harpooned whales that were only slightly wounded but that died a few minutes after surfacing [6]. One whale died after four to five breaths at the surface following a 230 m dive [6], although loss of the whale prevented confirmation of the cause of death. An experimentally dived harbour seal died in the procedure of being dropped to 300 m in 3 min and ascended in 9 min [6]. The necropsy showed an abundance of gas emboli in the mesenteric arteries (major arteries that supply the small and large intestines and pancreas), but this was carried out the following day, which would have allowed any supersaturated tissues to off-gas and form bubbles *in situ*. In addition, the forced dive may have prevented the seal from exhaling before the dive, which would be its normal behaviour.

**Figure 2.**
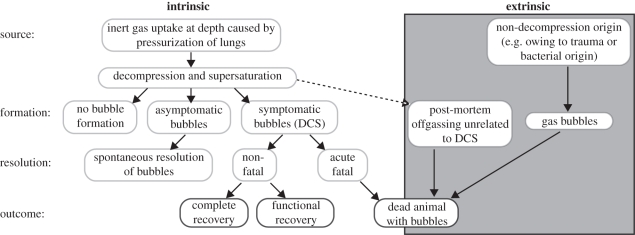
Potential scenarios for the formation and resolution of gas bubbles.

We suggest a range of scenarios that might pertain to gas-bubble formation and its potential consequences for marine mammals (figure 2). (i) Bubbles may be unrelated to decompression, arising via bacterial decomposition (putrefaction of tissues broken down by micro-organisms [36]) or via trauma (for example, magnetic resonance imaging (MRI) revealed cerebellar lesions in a young sea lion, probably resulting from arterial gas embolism following rib fracture [37]). (ii) Gas emboli may emerge from supersaturated tissue for several hours after death from an animal killed by another factor and then decompressed, as has been shown experimentally in sheep [38]. (iii) Gas emboli may be released from supersaturated tissue and contribute to the cause of death (i.e. DCS).

### (c) Gas kinetic models and tissue supersaturation

Models of gas kinetics are based on loading and unloading of N_2_ from lungs to the blood and body tissues [24,28,39,40]. While the results from these models should be viewed with caution and require verification, they provide useful working hypotheses for investigating which variables are possibly most important to understand this complex issue. Recent modelling has used increasingly complex compartmentalization scenarios, allowing differential N_2_ uptake at various tissues to reflect differential perfusion during diving, with some tissues (e.g. brain) having fast loading of N_2_ and other tissues (e.g. fat) having much slower loading. In fact, the circulatory adjustments during diving and the uptake of N_2_ by different tissues are poorly known for any diving vertebrate, so these models are limited to the sparse available data from a few species to account for tissue loading constants. Despite this, models of N_2_ loading have been fairly consistent in concluding that, under certain modelled diving conditions, tissues are likely to become supersaturated [28,39,40]. Some reports suggest that it is the shallower dives (prior to lung collapse) that have the greatest effect on the dynamics of N_2_ loading and unloading [39,41], whereas others argue that gas redistribution between tissues, owing to changes in perfusion, at depths beyond lung collapse must also be considered since slow tissues will still be loading at this stage, up to a maximum theoretical tension equal to the partial pressure of lung N_2_ gas at functional lung collapse [42,43]. Linking these models to measured dive profiles allows an examination of the predicted N_2_ tension during recorded diving behaviour and further demonstrates the possible supersaturation experienced at times by these marine mammals [43].

Differential gas uptake and clearance between fast and slow tissues, together with availability of gas nuclei, will cause variability in the presence of gas bubbles among different body tissues. In human free-divers, DCS, if present, is almost exclusively neurological, owing to fast ascent profiles [44] and/or vasoconstriction of peripheral tissues [24]. In contrast, modelling studies for marine mammals predict the highest end-dive supersaturation in the fatty tissues. Although these tissues are slow-loading, it is the short surface intervals between repeated dives that prevent them from fully off-loading, and they can thus accumulate higher long-term gas loads than fast-loading tissues [43,45]. This aligns with observation of cerebral ventricular, peribullar and jaw fat haemorrhages that were common factors among all beaked whale stranding reports [26], although whether such damage arose from endogenous gas bubbles is unknown.

## 3. Marine mammal diving physiology

The observations of bubbles and results of model predictions suggest that, even under normal diving conditions (figure 1), marine mammals may at times have N_2_ tensions sufficient to cause supersaturation at the surface despite their access to an extensive repertoire of adaptations to mitigate gas loading (electronic supplementary material, table S1). Thus, they appear either not to fully exploit these adaptations, or else at times they are not sufficient to avoid supersaturation. The main questions are, therefore, what causes such supersaturation and when and how this may become a significant health threat.

### (a) Diving adaptations and responses

The two primary diving adaptations thought to minimize N_2_ uptake at depth are the physiological ‘dive response’ and lung collapse. In fact, neither of these may be as comprehensive as first thought. Initial ‘forced submersion’ of captive animals [6] generated a maximum diving response (peripheral vasoconstriction and bradycardia). Recent studies using trained or naturally diving animals have shown similar responses, but these are reduced and/or more variable depending on the behaviour of the animal [10,46,47]. It is now known that the diving response varies with species, behaviour and maturity of the animal [11,46,48–50]. In addition, many elements of this response appear to be under cortical control, allowing their initiation prior to the onset of a dive [10,51,52].

Lung compression with diving may similarly not result in lung collapse depth as shallow as was initially suggested by the Scholander balloon-pipe model [6]. In fact, compression of the trachea will lead to a deeper lung collapse depth and progressive collapse of the alveoli will cause a graded decrease in diffusion [53]. Depths of lung collapse inferred from previous studies of blood N_2_ would be revised to deeper depths using this new model [3,53,54]. Experimental work exposing marine mammal cadavers with inflated lungs to imaging at pressure also suggests collapse depths deeper than previously assumed [55], although post-mortem changes in tissue compliance may have affected such results.

Several of the features thought to be adaptations common to marine mammals have been documented in relatively few species or in only certain species groups (electronic supplementary material, table S1). In the absence of more specific information, these results are sometimes assumed for other species: e.g. the assertion that pinnipeds are exhalation divers [56], when in fact this is only true for the phocid seals, some of which may in fact only reduce lung volume by 20 per cent [57]. Similarly, there are considerable differences in thoracic structural morphology [58] among cetaceans and pinnipeds, which together with these divergent inhalation behaviours at the onset of a dive should strongly caution against extrapolations across species.

### (b) Physiological trade-offs

**Figure 3.**
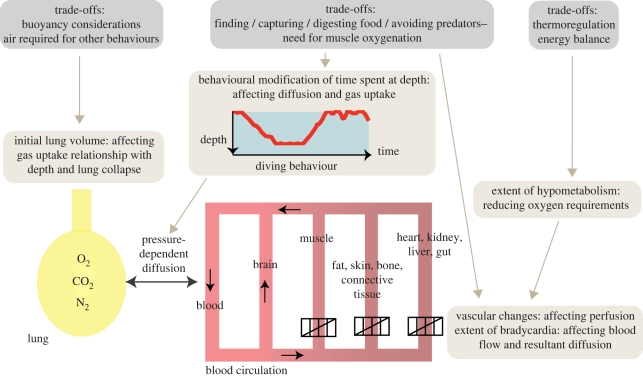
Selected mechanisms (in light grey) available to animals in managing gas loading (oxygen, O_2_; carbon dioxide, CO_2_; nitrogen, N_2_) between lungs and different body compartments, with the physiological trade-offs (in dark grey) that might influence these.

It seems likely that at any time, diving vertebrates are faced with a plethora of physiological challenges of which minimizing N_2_ absorption is only one. They manage N_2_ loading along with other necessities such as minimizing oxygen consumption, maximizing foraging success and avoiding predators, or homeostasis constraints, such as maintaining adequate core temperatures (figure 3).

In terms of gas dynamics, while reducing lung gas is beneficial for reducing the depth of alveolar collapse and thus reducing gas uptake at depth, some species (e.g. otariids, turtles, cetaceans and birds) may rely on lung oxygen stores while diving, or others may require air stored in the lungs for behaviours at depth (e.g. humpback whale bubble-netting to concentrate prey). Lung gas can have a large effect on the buoyancy of diving animals, and penguins have been shown to control the amount of inhaled air dependent on the depth of the subsequent dive [59]. Air at depth is also required for sound production (of either echolocation clicks or social calls) in cetaceans, although at deeper depths such air has less effect on buoyancy, and it may be stored in separate sinus and nasal cavities that do not support significant diffusion to the vascular system.

The dive profile, lung volume and pre-dive surface interval exhibited by animals are therefore likely to be a complex result of several physiological trade-offs. These could include optimizing buoyancy, maximizing foraging, avoiding predation, dealing with body maintenance functions (digestion and thermoregulation) or dealing with lactic acid build-up, while moderating N_2_ loading and maintaining O_2_ supply to obligate aerobic organs (figure 3). Evidence of such trade-offs has been observed in grey seals [60], which appear to defer the costs of digestion until extended surface intervals, and in northern elephant seals, which perform ‘drift’ dives for this purpose [61]. Similarly, other physiological trade-offs may include managing thermoregulation in addition to diving and exercising [62].

Models of diving behaviour initially focused on oxygen considerations [63], assuming that animals would dive on a maximally inhaled breath. Trade-offs were incorporated into these models in terms of maximizing foraging success based on depth and density of prey within the constraints of oxygen limitation [64] or of incorporating thermal and digestive constraints in analysis of foraging behaviour [65]. However, to our knowledge, such models have not yet considered diving behaviour in terms of the limitations caused by N_2_ gas kinetics alongside the simultaneous maximization of foraging success.

There is strong evidence that diving mammals have some control (voluntary or reflexive) over the intensity of the dive response [9,10,51,52] and that they can modify the response according to the trade-offs they face. The variability observed in heart rate [11,12,66], resting metabolic rate [67] and renal and hepatic blood flow [68], which are reduced as a function of dive duration, suggest some type of control over facets of the dive response, and, in turn, the potential for dramatic variations in tissue-specific N_2_ partial pressure. Similarly, there is evidence that in some situations marine mammals routinely exceed their presumed limits, e.g. exceeding their aerobic capacity and possibly tolerating a build-up of lactic acid to optimize foraging efficiency [41,69]. If such control is feasible for the dive response, diving mammals could potentially be allowing their levels of N_2_ saturation to increase occasionally in order to meet other targets. It might then be possible that certain preconditions of high saturation levels, combined with behavioural or physiological responses to a perceived threat, exacerbate saturation levels and lead to the appearance of bubbles. Although observations of potential decompression injury have thus far come from anthropogenic triggers, little is known regarding the perception and response to natural versus anthropogenic threats, and it is plausible that the same response could be triggered by rare natural events.

### (c) Bubble avoidance or tolerance

If marine mammals indeed live with N_2_ tensions at times much higher than previously supposed, it is possible that they have mechanisms to avoid bubble formation such that the threshold needed for supersaturation to lead to bubbles is much higher than in other species. Alternatively (or additionally), it may be that decompression-induced bubbles are relatively common but that marine mammals have unknown adaptations allowing them to tolerate these under natural conditions.

The presence of bubbles has been examined in two recent studies. A trained captive bottlenose dolphin monitored using Doppler and/or two-dimensional imaging ultrasound after a series of 10–12 dives (30–100 m depths) showed no evidence for vascular N_2_ bubble formation in either the portal or brachiocephalic veins [31]. However, the diving pattern of this animal was shallower and shorter than that of many wild marine mammals. A low level of bubble incidence has been detected in stranded (common and white-sided) dolphins via B-mode ultrasound. This appeared to be tolerated since most released animals suffered no obvious adverse consequences (i.e. they showed normal behaviour and did not restrand) [35]. No bubbles were detected in temporarily captured Sarasota Bay bottlenose dolphins [35], leading the authors to speculate that it may have been the inability to recompress that led to the appearance of bubbles in the stranded dolphins.

Our assessment of marine mammal diving stress is unavoidably influenced by our knowledge of human diving hazards, but the etiology of diving injury in marine mammals could potentially be quite different. Given the differences between terrestrial and marine mammals, and even between marine mammal species, in terms of diving behaviour, physiology and anatomy (electronic supplementary material, table S1), it may be simplistic to assume that the presentation of DCS would be identical to humans'. Similarly, there may be different sensitivities to bubble presence between terrestrial and marine mammals, and even between different marine mammal species or lineages [70], leading to differences in risk between species.

## 4. Future research

This paper summarizes the discussion and debate generated during a workshop convened to review the current state of knowledge of marine mammal gas kinetics. The integration of disparate scientific communities spanning human diving medicine, veterinary pathology, comparative animal anatomy, physiology, ecology and behaviour was crucial to this discussion and such interdisciplinary work is likely to greatly facilitate future research in this field.

The discussions provided a critical and up-to-date analysis of our current understanding, drawing, on aspects across this broad range of disciplines. Arising from this, we highlighted three main themes in terms of future research avenues (table 1): (i) diving physiology and responses, (ii) diving behaviour and bubble incidence, and (iii) bubble avoidance, tolerance, effects and pathophysiology. The first theme concerns the drivers causing supersaturation and bubble formation. Our understanding of diving responses, including the depth and mechanism of lung collapse and the changes to blood flow (and resulting changes to N_2_ uptake and removal) during diving, is based on few studies of few species. We suggest several methods to investigate additional specifics of the dive response. The second theme concerns how common bubbles are in conjunction with variations in diving behaviour. In terms of relating bubbles to behaviour, this can be done post hoc, relating information collected at strandings to broad generalizations about average species diving behaviour. Alternatively, development of methods for bubble detection coincident with measurements of diving behaviour (subjected to both natural and anthropogenic novel threats) would allow greater resolution of the connection between behaviour and bubble incidence. The third theme concerns understanding the circumstances under which bubbles generate a significant threat. A comprehensive documentation of strandings will enable description of the presence and distribution of bubbles in stranded cadavers. At the cellular level, we know little about how bubbles cause sub-lethal harm, whether there is an immune response to bubble formation, and how marine mammals differ from terrestrial mammals in their reaction to bubbles.

**Table 1.** Future research priorities and potential techniques to address these research avenues.

topic	specific research	potential methods
diving physiology and responses	mechanics of lung collapse	hyperbaric pressure chamber work with small marine mammals
	kinetics of N_2_ uptake and distribution	respiratory gas analysis and blood and tissue measurement, aided by techniques such as Van Slyke, mass spectrometry and gas chromatography
	gas dynamics at the alveolar boundary	alveolar and arterial gas sensors
	soft-tissue changes (alveolar collapse) and shunting of blood	medical imaging (ultrasound, CT and MRI); potential use of polarized gas as more successful contrast agent
	passive (pressure-induced) changes to the circulatory system with lung compression?	rubberized casts of the circulatory system at ambient and elevated pressures
	perfusion patterns in terms of vascular anatomy and pathology	conventional or CT angiography
	changes in blood flow distribution during diving	use of a radioactive isotope of inert gas (e.g. Xe^127^ or Xe^133^) with small external gamma ray sensors on the body surface
diving behaviour and bubble incidence		
	comparison of bubble incidence with diving behaviour	consistent, replicable protocols for strandings nationally and internationally
	detection of bubbles and measurement of local blood flow	intra-vascular ultrasound catheter
	measurement of extravascular bubbles from free-swimming animals	development of dual-frequency ultrasound incorporated into attached bio-logging tag
	bubble incidence in other high-stress situations including novel anthropogenic or natural threats	physiological monitoring during novel stimulation in shallow and deep divers
bubble avoidance, tolerance,	bubble gas composition	gas sampling
effects and pathophysiology	are bubbles more likely to occur and be fatal in certain tissues?	distribution of bubbles in stranded cadavers
	how do bubbles cause sub-lethal harm? is this via an immune response?	effect of bubbles on *in vitro* cell cultures; cellular and molecular differences between marine and terrestrial mammals in terms of reaction to bubbles

## 5. Conclusion

Under most natural conditions, diving vertebrates appear to dive without bubble-induced decompression injury. However, the evidence suggests that they may deal with the precursors to this, i.e. supersaturation and bubble presence, on a more regular basis than previously thought. It seems that the physiological adaptations that mitigate N_2_ loading during dives are not predetermined responses that prevent or minimize N_2_ loading, but rather could be modified, as needed, on a dive-by-dive basis according to other trade-offs, thus resulting in greater variation in blood N_2_ levels than was previously hypothesized. Our view of marine mammal adaptations should therefore change from one of simply *minimizing N_2_ loading* to one of *management of the N_2_ load*. We suggest that variability in management of N_2_ may be required as divers are faced with several physiological trade-offs within their diving behaviour. It is then possible that a response to an unanticipated acute threat (such as man-made noise) perceived as more immediately critical than management of N_2_ might result in decompression injury. This may be strategic risk-taking regarding N_2_ load on the part of the animal but could nevertheless prove ultimately deleterious.
